# Pancreatic liposarcoma: A case series and literature overview of an extremely rare soft tissue tumor

**DOI:** 10.1016/j.ijscr.2025.111699

**Published:** 2025-07-18

**Authors:** Zahraa M. Alhulaili, Robbert J. de Haas, Arjen Cleven, Barbara L. van Leeuwen, Maarten W. Nijkamp, Joost M. Klaase

**Affiliations:** a University Medical Center Groningen, Department of Hepato-Pancreato-Biliary Surgery and Liver Transplantation, Groningen, the Netherlands; b University Medical Center Groningen, Department of Radiology, Groningen, the Netherlands; c University Medical Center Groningen, Department of Pathology, Groningen, the Netherlands; d University Medical Center Groningen, Department of Surgical Oncology, Groningen, the Netherlands

**Keywords:** Pancreas, Pancreatic liposarcoma, Pancreatoduodenectomy, Complications, Aerobic fitness

## Abstract

**Introduction and importance:**

Liposarcoma is a subtype of soft tissue sarcoma. It can originate from any adipose tissue in the body. However, the most common sites are deep soft tissues of the lower extremities and retroperitoneum. Liposarcoma arising from the pancreas is extremely rare. It occasionally presents with epigastric pain and abdominal distension, yet, in most cases it is asymptomatic. It can be discovered incidentally on radiological imaging. Complete surgical excision is the only effective cure. Definitive pathological classification can be made after histopathological assessment with additional diagnostic features in biopsy material of the resected tumor. Herein, we present a case series of three consecutive adult patients with pancreatic liposarcoma who were treated surgically to increase our understanding of surgical and pathological aspects of this type of tumor.

**Case presentation:**

The first patient is a 75-year-old female with recurrence of a well differentiated liposarcoma with partial dedifferentiation in the pancreatic head who was managed surgically with pylorus-resecting pancreaticoduodenectomy (PrPD). The second patient is an 80-year-old female with pleomorphic liposarcoma of the pancreatic head who was managed surgically with the same approach. The final patient is a 72-year-old male with primary dedifferentiated liposarcoma and well-differentiated liposarcoma located dorsal to the duodenum, in close contact with the right renal fascia and with ingrowth into the pancreatic head. This patient was managed surgically with PrPD involving right nephrectomy.

**Clinical discussion:**

In this case series, we added three new cases of pancreatic liposarcoma to twenty earlier reported cases in the literature. Complete resection is not the only indicator of recurrence, recurrence can be faster in high grade tumors, such as dedifferentiated liposarcoma and pleomorphic liposarcoma. None of the patients in our study underwent (neo)-adjuvant chemo-radiotherapy.

**Conclusion:**

Surgical resection remains the curative treatment of choice for pancreatic liposarcoma. Pancreatoduodenectomy is considered safe, and it should only be performed when in line with patient physical fitness and preferences. Personalized exercise program to improve fitness is recommended in patients with low aerobic fitness.

## Highlights

• We presented a case series of three consecutive adult patients with pancreatic liposarcoma treated surgically at our institution
• We made a descriptive overview of the available literature on comparable 20 cases
• Our case series represents the third and the fourth cases of coexisting well differentiated liposarcoma and dedifferentiated liposarcoma and the fifth case of pancreatic pleomorphic liposarcoma.
• Surgical resection remains the curative treatment of choice. Pancreaticoduodenectomy is considered safe, and it should only be performed when in line with patient physical fitness and preferences. Personalized exercise program to improve fitness is recommended in patients with low aerobic fitness.

## 1 Introduction

Liposarcoma is a subtype of soft tissue sarcoma that arises from adipocytes [1]. It has a prevalence rate between 15 and 20 % of all soft tissue sarcomas [2]. It can occur at any age, yet most commonly in patients aged between 60 and 70 years old [3]. According to the 2020 World Health Organisation (WHO) classification, adipocytic tumors can be classified into three main categories namely, benign, intermediate, and malignant [1]. Pending on their pathological and molecular profile, liposarcomas can be further subdivided according to their histopathological profiles into well differentiated liposarcoma (WDLPS), pleomorphic liposarcoma (PLPS), dedifferentiated liposarcoma (DDLPS), myxoid liposarcoma (MLPS), and myxoid pleomorphic liposarcoma (MPLPS) [1]. Liposarcomas can arise from any fatty tissue in the body [3]. The most common sites are, however, deep soft tissues of the lower extremities followed by retroperitoneum [4]. Retroperitoneal sarcomas account for less than 10 % of all sarcoma cases with an incidence rate of 2.7 per million people annually [5,6]. Complete surgical resection is the cornerstone of treatment for retroperitoneal sarcoma, however in recent years the additional value of (neo)adjuvant (chemo) radiation is a subject of debate [7,8]. Five-year survival following complete resection is 41–50 % and the disease often recurs [4,5].

The most common subtype of pancreatic tumor originates from epithelial tissues, such as pancreatic ductal adenocarcinoma (PDAC) which accounts for 85 % of all pancreatic malignancies [2]. Liposarcomas arising from the pancreas are extremely rare occurring in less than 1 % of all pancreatic tumor cases [4]. A recent literature search revealed 20 cases of pancreatic liposarcoma reported in the English literature since 1979 as shown in Table 1 [[2], [3], [4],[8], [9], [10], [11], [12], [13], [14], [15], [16], [17], [18], [19], [20], [21], [22], [23], [24]]. Pancreatic liposarcoma occasionally presents with epigastric pain and abdominal distension [2]. Other symptoms, such as jaundice, loss of appetite and weight loss may occur as well, based on location of the tumor [2]. Yet, in most cases it is asymptomatic and it can be discovered incidentally on radiological imaging, including ultrasonography (US), computed tomography (CT) and magnetic resonance imaging (MRI).

**Table 1:** Previously reported cases of pancreatic liposarcoma.

Case study ID	Gender	Age	Main complaint	Location of liposarcoma	Liposarcoma type	Treatment	Additional management	Recurrence/prognosis
Machado et al. [2]	Male	42	Abdominal pain	Pancreatic head, neck	Dedifferentiated liposarcoma with pleomorphic and myxoid liposarcoma	Distal pancreatectomy and splenectomy	Adjuvant chemotherapy and radiotherapy	No recurrence after 5 years
Tanabe et al. [3]	Female	81	Asymptomatic	Pancreatic tail	Dedifferentiated with well differentiated liposarcoma	Laparoscopic distal pancreatectomy and splenectomy	No	Not reported
Wilson et al. [4]	Male	52	Asymptomatic	Pancreatic tail with invasion into the spleen, gastric wall, and left colon wall	Dedifferentiated liposarcoma	Radical resection including distal pancreatectomy, partial gastrectomy, splenectomy, left hemicolectomy, and left adrenalectomy	Adjuvant chemotherapy with Pembrolizumab	No recurrence after 12 months
Kuramoto et al. [9]	Male	24	Abdominal distension	Pancreatic body	Myxoid	Resection pancreatic body	No	Recurrence after 44 months, managed with resection
Dodo et al. [10]	Male	76	Abdominal pain and weight loss	Pancreatic body, tail and involvement of spleen and duodenum	Well differentiated with area of dedifferentiated liposarcoma	Distal pancreatectomy and splenectomy	Adjuvant radiotherapy	No recurrence after 26 months
Elliott et al. [11]	Female	59	Abdominal distension	Whole pancreas except 3 cm from ampulla of Vater	Pleomorphic	Pancreatectomy and splenectomy	No	No recurrence after 6 years
Cao et al. [12]	Female	72	Asymptomatic	Pancreatic body, tail with contact to the spleen, left adrenal gland	Well differentiated liposarcoma	Distal pancreatectomy, splenectomy, and left adrenalectomy	No	Not reported
Han et al. [13]	Female	29	Abdominal distension and vomiting	Pancreatic tail	Dedifferentiated liposarcoma	Distal pancreatectomy and splenectomy	Adjuvant chemotherapy epirubicin and ifosfamide	Recurrence 6 months after the operation managed with apatinib and paclitaxel
Matthews et al. [14]	Female	65	Asymptomatic	Pancreatic tail	Well differentiated liposarcoma	Unspecified surgical resection	No	Not reported
Kim et al. [15]	Female	78	Asymptomatic	Pancreatic body	Well differentiated liposarcoma	Complete excision	Adjuvant chemotherapy	Not reported
Liu et al. [16]	Female	28	Abdominal pain	Pancreatic tail	Dedifferentiated liposarcoma	Distal pancreatectomy with splenectomy	No	No recurrence after 26 months
Xu et al. [17]	Male	69	Abdominal and back pain	Uncinate process of pancreas	Combination of dedifferentiated, myxoid and pleomorphic	Pancreatoduodenectomy with complete excision and lymphadenectomy	Adjuvant chemotherapy and radiotherapy	No recurrence after 10 months
Puspanathan et al. [8]	Female	49	Obstructive jaundice	Pancreatic head	Pleomorphic	Pancreatoduodenectomy	No	Not reported
Carboni et al. [18]	Male	66	Asymptomatic	Metastasis from left lower extremity to pancreatic head	Myxoid liposarcoma	Pancreatoduodenectomy	Complete pancreatectomy due to haemorrhage	No recurrence after 6 months
Huang et al. [19]	Male	57	Abdominal distention, vomiting and weight loss	Left kidney invading the pancreatic body	Myxoid liposarcoma	Unspecified surgical resection	Reoperation due to abdominal distention and vomiting with a mass on the CT scan	Reoperation in 10 days due to residual tumor, died during the operation due to sudden cardiac death
Yue et al. [20]	Female	52	Abdominal pain	Pancreatic head	Pleomorphic	Unspecified surgical resection	No	No recurrence after 12 months
Xiang et al. [21]	Female	65	Asymptomatic	Pancreatic body	Dedifferentiated liposarcoma	Resection of the pancreatic body and tail and spleen	No	After 2 months metastases in the liver, stomach, retroperitoneum, and remnant pancreas.
Malleo et al. [22]	Male	30	Asymptomatic	Metastasis from the right axilla to pancreatic body and tail	Pleomorphic Liposarcoma	Distal pancreatectomy with splenectomy	No	No recurrence after 20 months
Wang et al. [23]	Female	40	Asymptomatic	Metastasis from right thigh to pancreatic body	Myxoid liposarcoma	Medial pancreatectomy and pancreaticojejunostomy	Recurrence tumor right thigh resected and followed by radiotherapy	No recurrence after 22 months
Abe et al. [24]	Male	63	Vomiting and anorexia	Metastasis from retroperitoneum to pancreatic body and gastric cancer	Dedifferentiated liposarcoma	No resection	Chemotherapy with doxorubicin and ifosfamide	Bone metastases after 10 months
Present study patient 1	Female	75	Back and abdominal pain, nausea without vomiting and a single episode of syncope	Pancreatic head	Recurrence of well differentiated liposarcoma and dedifferentiated liposarcoma	Pylorus-resecting pancreaticoduodenectomy (PrPD)	No	Renal cell carcinoma after 6 months
Present study patient 2	Female	80	Asymptomatic	Pancreatic head	Pleomorphic liposarcoma	Pylorus-resecting pancreaticoduodenectomy (PrPD)	No	Liver metastases after 6 months. Died 28 months postoperatively.
Present study patient 3	Male	72	Abdominal pain	Dorsal to duodenum in close contact with the right renal fascia and with ingrowth into the pancreatic head	Primary well differentiated liposarcoma and dedifferentiated liposarcoma	PrPD, en bloc resection of the right anterior renal fascia, right nephrectomy, and appendiceal base	No	No recurrence after 18 months

In this study, we present a case series of three consecutive adult patients with pancreatic liposarcoma treated surgically at our institution in combination with a descriptive overview of the available literature on comparable cases.

## 2 Methods

This is a single academic center retrospective case series study. Data of three consecutive adult patients with pancreatic liposarcoma who were treated surgically between 2021 and 2024 at our hospital were retrieved from our database. This study has been reported in line with the PROCESS criteria [25].

## 3 Statement of ethics

This case series was conducted in accordance with the applicable Dutch laws. There was no need for ethical approval from the local committee of our hospital according to the institutional requirements. Patients in this case series were selected from our hospital database for pancreatic surgery. All patients who underwent pancreatic surgery since 2018 received a prehabilitation program and were registered in the FRAIL study under the registration number 201800293. Informed consent was obtained from all participants.

## 4 Case presentation

### 4.1 Case 1

**Fig. 1:**
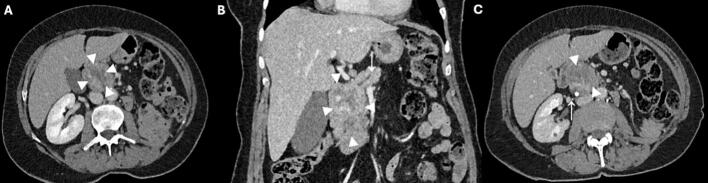
Portal venous phase abdominal CT in axial (A, C) and coronal (B) direction, showing a mass (between arrowheads) in the pancreatic head with a maximum diameter of 37 mm and heterogeneous enhancement. Also, the pancreatic duct proximal to the mass is dilated (arrow in image B). (C) showing the mass (between arrowheads) in the pancreatic head with a maximum diameter of 43 mm and heterogeneous enhancement. A stent in the common bile duct is present (arrow).

**Fig. 2:**
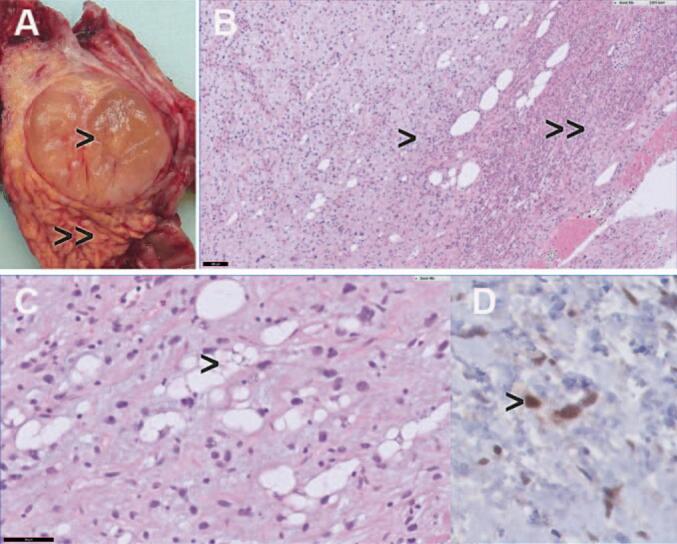
Histopathological findings in patient 1. (A) resected pancreatic liposarcoma with permeative growth in the pre-existing pancreas (arrowhead = liposarcoma; double arrowhead = pre-existing pancreatic tissue. (B—C) Hematoxylin-eosin staining showing liposarcoma with lipoblast and pleomorphic spindle cells (arrowhead) with permeative growth into pancreatic tissue (double arrowhead) (D) Positive MDM2 nuclear immunostaining in tumor cells indicative for MDM2 amplification. These findings are consistent with dedifferentiated liposarcoma (DDLPS) and well-differentiated liposarcoma (WDLPS).

This patient concerned a 75-year-old woman with a medical history of Lyme disease, hypertension, right ovarian cyst and a well differentiated left retroperitoneal liposarcoma which was resected involving left nephrectomy. The patient presented four years after nephrectomy to her general practitioner with episodes of back and abdominal pain that were associated with nausea and a single episode of syncope. An abdominal ultrasound (US) revealed a 30 mm mass in the pancreas. Subsequently, an abdominal CT scan was performed that demonstrated a tumor of 37 mm in the pancreatic head, suspicious for a malignancy as shown in Fig. 1 **(A, B)**. The patient was referred to the department of Hepato-Pancreato-Biliary surgery at our hospital where the decision to perform a pylorus-resecting pancreatoduodenectomy (PrPD) was made after a discussion by a multidisciplinary team. There was no need for preoperative biopsy since a biopsy would not have changed the treatment plan. The patient was active, she walked 30 minutes a day five times a week and cycled regularly. The cardiopulmonary status of the patient was evaluated preoperatively using cardiopulmonary exercise testing (CPET) that showed a ventilatory anaerobic threshold higher than 11 ml/kg/min. Therefore, there was no indication for extra physical training. In addition, as part of the standard evaluation lung function test was performed, and the results were within the normal range. The waiting time for the surgery was 9 weeks due to logistical reasons and the patient was advised to stay physically active. During this time, the pancreatic lesion showed a slight growth to 43 mm Fig. 1 **(C)**. Two weeks before the surgery, the patient developed symptoms of itching, jaundice, and stool discoloration. These symptoms were related to biliary obstruction and managed with endoscopic retrograde cholangiopancreatography (ERCP), papillotomy and the placement of a plastic stent in the common bile duct Fig. 1 **(C).** The patient underwent a PrPD without vascular resection. Histopathological examination of the resected specimen revealed tumor metastases in the pancreas of the previously resected retroperitoneal liposarcoma. It demonstrated both well differentiated liposarcoma (WDLPS) and dedifferentiated liposarcoma (DDLPS). Fig. 2. No metastases were present in the resected lymph nodes, and no malignancy was found in the gallbladder or hepatic hilar lymph nodes. The resection margins around the uncinate process were clear of tumor. The postoperative period was complicated with hyponatraemia caused by the syndrome of inappropriate antidiuretic hormone release (SIADH) which was treated with fluid restriction. The patient was discharged two weeks after the surgery, and she was scheduled for regular follow-up at our hospital. She did not undergo any adjuvant chemo-radiotherapy. In the first three postoperative months, nutritional intake was disturbed due to frequent nausea and vomiting which has improved over time. Six months after the operation upon regular follow-up, the abdominal MRI showed no recurrence of liposarcoma. Nevertheless, a new mass of 9.7 mm was seen in the right kidney that was suspicious for renal cell carcinoma. Notably, the same lesion was present in the CT scan performed two years earlier and it was 5 mm. In addition, approximately 20 % of the small kidney tumors that are smaller than 40 mm is benign [26,27]. The patient case was discussed by a multidisciplinary team. The patient was then referred to the urology department for further management. The treatment options were discussed which included active surveillance, ablation and lesion excision. The patient opted for active surveillance policy since the tumor grows slowly. The active surveillance policy involved regular follow-up visits, initially every 2 months with echography and blood test, and later every 6 months with a CT scan and blood test. Until today one year after the operation, the patient reported to have a good quality of life, she lives independently and engages in physical activities regularly.

### 4.2 Case 2

**Fig. 3:**
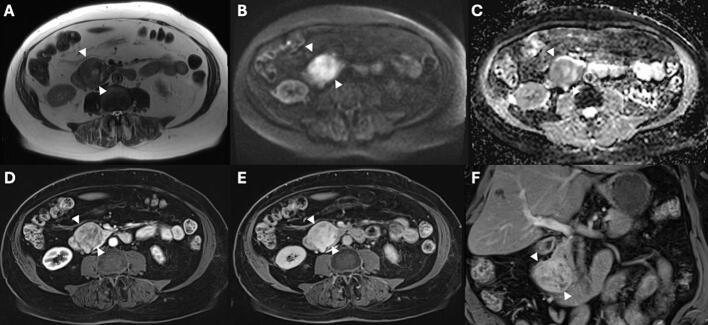
Abdominal MRI in axial (A-E) and coronal (F) direction. A mass in the pancreatic head (arrowheads) with a maximum diameter of 49 mm is observed, with a heterogeneous hyperintense signal intensity on T2 weighted imaging (A), and clear diffusion restriction (B and C), as well as a heterogeneous enhancement pattern in the late arterial (D) and portal venous phase (E and F).

**Fig. 4:**
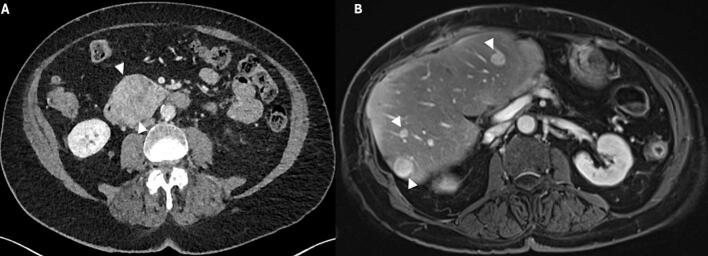
(A) Portal venous phase abdominal CT in axial direction, showing a mass (between arrowheads) in the pancreatic head with a maximum diameter of 57 mm and heterogeneous enhancement. (B) Contrast-enhanced abdominal MRI in axial direction in the portal venous phase, showing several enhancing nodular lesions (arrowheads) diffusely distributed in the liver, suspicious for liver metastases.

**Fig. 5:**
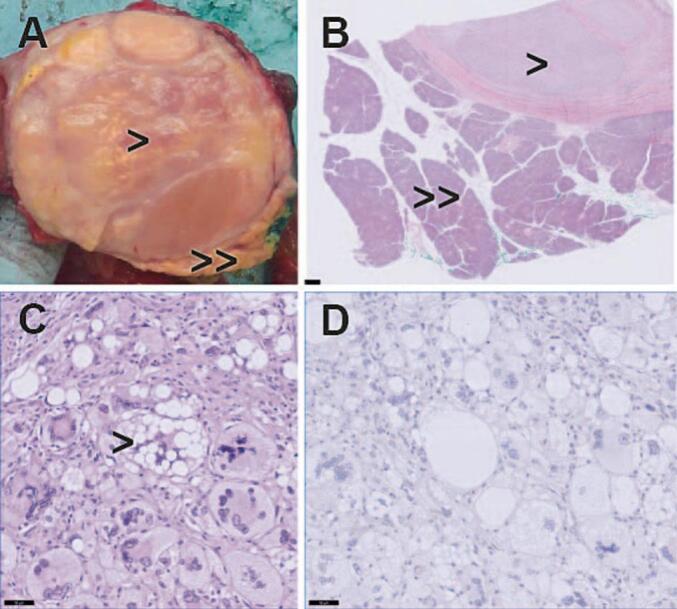
Histopathological findings in patient 2. (A) resected pancreatic liposarcoma with a diameter of 62 mm (Arrowhead = liposarcoma; double arrowhead = normal pancreatic tissue) (B—C) Hematoxylin-eosin staining shows pleomorphic lipoblasts (arrowhead). (D) No expression of MDM2 therefore well- differentiated and dedifferentiated liposarcomas were ruled out.

An 80-year-old woman with a medical history of hypertension and right sided renal cell carcinoma was treated with partial nephrectomy. Five years later upon follow-up the ultrasound of the kidney revealed a lesion in the pancreas. Further diagnostics with MRI showed a mass of 49 mm in the pancreatic head, without evidence of liver metastases Fig. 3. Endoscopic ultrasound (EUS) failed initially due to low oxygen saturation. The chest-abdominal CT scan that was performed one month later revealed a large mass of 57 mm in the pancreatic head as illustrated in Fig. 4 **(A)**. A core biopsy of the lesion was then successfully obtained through EUS. The cytology outcome was most suspicious for a primary tumor of the pancreas. The patient was active with one hour walks four times per week. The cardiopulmonary status of the patient was preoperatively evaluated using cardiopulmonary exercise testing (CPET) that showed a ventilatory anaerobic threshold more than 11 ml/kg/min. Therefore, there was no indication for extra training, but she was advised to stay physically active. The patient had clinical frailty score 2 [26]. In addition, as part of the standard evaluation lung function test was performed, and it was slightly restrictive. She underwent PrPD without vascular resection. The postoperative period was complicated by prolonged delayed gastric emptying grade C according to the International Study Group for Pancreatic Surgery (ISGPS) guidelines, which was managed conservatively with a nasogastric tube [28]. Histopathological examination of the resected specimen revealed pleomorphic liposarcoma (PLPS) with a maximum diameter of 62 mm Fig. 5 **(A-C)**. A conventional well differentiated or dedifferentiated liposarcoma with MDM2 amplification was ruled out using immunohistochemistry showing no expression of MDM2 Fig. 5 **(D)**. Angioinvasive growth was focally suspected, and there were no lymph node or distant metastases. The patient did not undergo any neoadjuvant or adjuvant chemo-radiotherapy. The patient was discharged 3 weeks after the surgery, and she was scheduled for regular follow-up at our hospital. The patient developed diabetes mellitus (DM) postoperatively which was managed with insulin. Six months later, upon regular follow-up, the abdominal CT scan showed multiple hypodense lesions in the liver suspicious for metastases. An MRI was performed which confirmed metastatic disease as shown in Fig. 4 **(B)**. At that time, there were no curative treatment options anymore. The patient case was discussed in a multidisciplinary team, and she was considered a candidate for palliative systemic therapy. The patient, however, refused further treatment because quality of life was important to her, and she did not want to visit the hospital regularly. The patient lived two good years after the operation. After that, her condition began to deteriorate, and she died 28 months after surgery.

### 4.3 Case 3

**Fig. 6:**
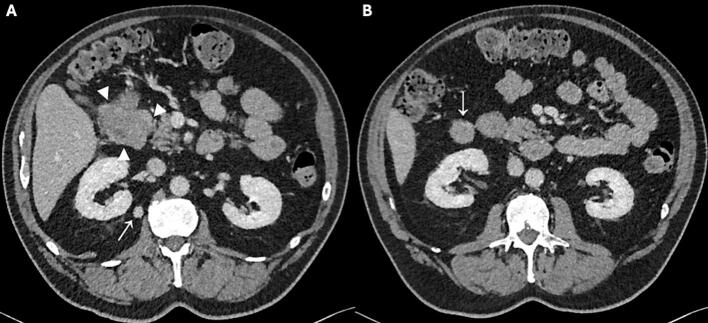
Portal venous phase abdominal CT in axial direction, showing a mass (between arrowheads in A) in the proximal duodenum with a maximum diameter of 56 mm and slight enhancement. Besides, two nodular lesions at the anterior and medial fascias of the right kidney are present (arrows in A and B).

**Fig. 7:**
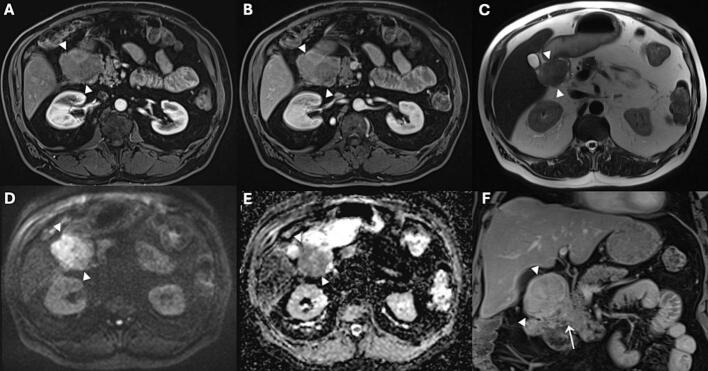
Abdominal MRI in axial (A-E) and coronal (F) direction. A mass in the proximal duodenum (arrowheads) is visible, with only slight heterogeneous enhancement in the late arterial (A) and portal venous phase (B and F), heterogeneous slightly hyperintense signal intensity on T2 weighted imaging (C), and clear diffusion restriction (D and E). The lesion extends into the fatty tissue surrounding the duodenum as well as in the pancreaticoduodenal groove (arrow in F).

**Fig. 8:**
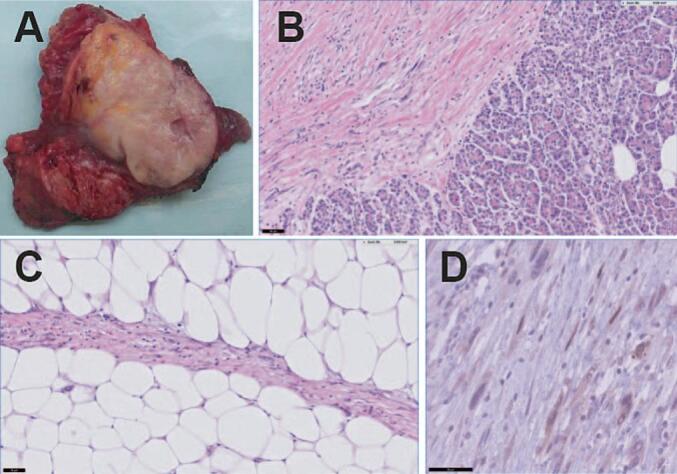
Histopathological findings in patient 3. (A) resected pancreatic liposarcoma with a diameter of 95 mm. (B) Hematoxylin-eosin staining shows spindle-shaped tumor cells (left upper part) infiltrating pre-existing pancreatic glandular tissue (right lower part). (C) Fibrous septae with atypical hyperchromatic spindle cells surrounded by abundant lipogenic component with lipoblasts (D) The tumor cells showed strong nuclear MDM2 staining indicative for MDM2 amplification. These findings are diagnostic for dedifferentiated liposarcoma (DDLPS) and well-differentiated liposarcoma (WDLPS).

A 72-year-old man with a history of nicotine and alcohol abuse, hypertension, insulin dependent type 2 diabetes mellitus (DM), hepatic steatosis and peripheral vascular disease was endoscopically treated at our hospital for adenoma of the appendix with low grade dysplasia. Nine months later an abdominal CT scan was performed because of persistent abdominal pain. The scan revealed no abnormalities around the appendix area, yet it unexpectedly showed a growing lesion at the site of the right anterior renal fascia, right pararenal area and at the site of the proximal duodenum, as shown in Fig. 6, suspicious for malignancy. Additional investigation of the lesions consisted of gastroscopy and EUS with biopsy of the lesion. The gastroscopy showed a large lesion of approximately 18 × 29.5 mm in the duodenal bulb immediately distal to the pylorus. Subsequently, CT guided biopsy was performed, and it disclosed multifocal intraperitoneal dedifferentiated liposarcoma (DDLPS). To evaluate the extension of the lesion an MRI was performed, and it showed high-grade liposarcoma dorsal to the duodenum in contact with the pancreatic head, gastroduodenal artery and growing into the duodenum as illustrated in Fig. 7. The patient was active, and he walked two times a day for 45 minutes and cycled 40 minutes in the weekend. The cardiopulmonary status of the patient was preoperatively evaluated using cardiopulmonary exercise testing (CPET) that showed a ventilatory anaerobic threshold higher than 11 ml/kg/min. Therefore, there was no indication for extra training. However, the patient was advised to stay physically active. In addition, as part of the standard evaluation lung function test was performed, and it showed a normal result. The patient underwent open PrPD, en bloc resection of the right anterior renal fascia, right nephrectomy, and appendiceal base resection in view of irradicality of a previous adenoma in the appendix. On postoperative day 3 (POD3), the patient had high drain amylase level of 1.356 U/L. The postoperative period was complicated by delayed gastric emptying grade C according to the ISGPS definition, postoperative pancreatic fistula grade B according to the ISGPS guidelines treated with drain placement and antibiotics, clostridium colitis treated with antibiotics, and wound infection [29]. Histopathological examination of the resected specimen revealed dedifferentiated liposarcoma (DDLPS) and well differentiated liposarcoma (WDLPS) with a maximum diameter of 95 mm Fig. 8 **(A-C)**. Using immunohistochemistry, the tumor cells showed strong nuclear MDM2 staining indicative for MDM2 amplification Fig. 8 **(D)**. The resection margins were tumor free. The patient did not undergo any neoadjuvant or adjuvant therapy. Upon discharge, the patient was scheduled for regular follow-up visits at our hospital according to local protocol. The patient presented himself one week after discharge with fever and general malaise. Laboratory results showed high infection parameters. An abdominal CT scan was performed, and an intra-abdominal fluid collection was found which was managed with a percutaneously placed pigtail drain. The drain culture showed *Streptococcus anginosus* which was managed with antibiotic. The patient had an overall complication grade of IIIa as defined by Clavien-Dindo; which means that the patient had an intervention that did not require general anaesthesia, in this case the patient had CT-guided drainage of the fluid collection under local anaesthesia [30]. In addition, the patient continued to experience cramping abdominal pain regularly and iron deficiency anemia. Due to persistence of these symptoms, biopsies were obtained 14 months postoperatively from the stomach and distal duodenum through gastroscopy which showed no further abnormalities. Until today, 18 months after surgery the patient is fit and there are no signs of disease recurrence or distant metastasis.

## 5 Discussion

Pancreatic liposarcoma is extremely rare. It is classified into different subtypes. Well differentiated liposarcoma and low grade myxoid liposarcoma (no round cell component) are low-grade tumors that tend to grow slowly and have low risk of metastases, whereas pleomorphic liposarcoma and dedifferentiated liposarcoma are high-grade tumors that tend to grow rapidly and are associated with poor prognosis and higher risk of metastases [4]. A transition from low-grade well differentiated liposarcoma into high-grade dedifferentiated liposarcoma arises in 10 % of all cases [4]. Dedifferentiated liposarcoma is however less aggressive compared to pleomorphic liposarcoma [4].

Our case series represents a patient with liposarcoma recurrence in the pancreas consisting of coexisting well differentiated liposarcoma and dedifferentiated liposarcoma who underwent a retroperitoneal tumor resection and left nephrectomy due to well differentiated liposarcoma of the left retroperitoneum four years earlier. The second patient with pleomorphic liposarcoma (PLPS) of the pancreatic head had liver metastases within six months after the resection of the pleomorphic liposarcoma. This is line with other publications indicating that pleomorphic liposarcoma is more aggressive with higher risk of metastases compared to conventional well or dedifferentiated liposarcoma with MDM2 amplification [4]. The five-year survival rate of pleomorphic liposarcoma is reported to be 18 % [16]. Notably, this patient had undergone partial nephrectomy due to right sided renal cell carcinoma five years prior to the diagnosis. The final patient had dedifferentiated liposarcoma and well differentiated liposarcoma. Histologically, well differentiated liposarcoma is characterized by the presence of unusual lipoblasts and thick fibrous septations, whereas dedifferentiated liposarcoma is characterized by the presence of spindled tumor cells and condensed fibrous stroma without prominent lipoblast [4]. As the tumor grows, lipoblasts become less visualized.

The initial diagnosis of all three patients was made by CT scan. In liposarcomas, CT imaging demonstrates heterogeneous low-density areas suggesting the presence of fat, often accompanied by more solid components [2,3]. While complete resection is proven to be the only effective cure for liposarcoma, a study by Liu et al. suggested that chemotherapy should be considered during the management of patients with pancreatic liposarcoma [16]. Elliott et al. indicated that adjuvant radiotherapy is recommended when surgical excision is incomplete. Adjuvant radiotherapy could induce longer periods of remission in case of incomplete resection, yet there is no additional advantage of radiotherapy if the tumor is totally resected [11]. In the previously published liposarcoma studies, four patients were treated with surgical resection followed by adjuvant chemotherapy, two patients with a combination of surgical resection and adjuvant chemo-radiotherapy and two patients with surgical resection and adjuvant radiotherapy. In our case series, all three patients underwent radical resection with free surgical margins and none of them underwent neoadjuvant or adjuvant chemo-radiotherapy. Complete excision of the tumor should also include the tumor capsule and nearby organs when needed [31]. Complete resection is not the only indicator of recurrence, recurrence can be faster in high grade tumors, such as dedifferentiated liposarcoma and pleomorphic liposarcoma [32]. In our case series the patient with a well differentiated left retroperitoneal liposarcoma had recurrence four years later of well differentiated liposarcoma and dedifferentiated liposarcoma in the pancreatic head.

Moreover, all patients in this case series underwent pylorus-resecting pancreatoduodenectomy (PrPD). In this operation the pyloric ring of the stomach is resected while preserving approximately 95 % of the stomach [33,34]. Studies have shown a decrease in the incidence of delayed gastric emptying (DGE) in patients who underwent PrPD compared to pylorus-preserving pancreatoduodenectomy (PPPD) which involved preservation of the pylorus [33,34]. Two of the three patients in this case series had DGE after PrPD. One of those patients had DGE grade B and the other patient had DGE grade C according to the ISGPS guidelines [28]. This could be due to presence of some risk factors in our patients, such as advanced age, smoking history, high American Society of Anaesthesiology (ASA) score, opioid use and pancreatic fistula grade B [35,36].

All three patients in our study were physically fit and functioned independently in their daily lives. Low oxygen uptake is associated with increased risk of morbidity and mortality especially after a major abdominal surgery such as pancreatoduodenectomy [37]. Cardiopulmonary exercise testing (CPET) was performed on all patients, and it showed an adequate ventilatory anaerobic threshold (VAT) above 11 ml/kg/min. This cut-off was used in the majority of studies concerning non-cardiac major surgery, whereby patients with a lower threshold were prone to postoperative morbidity and mortality [[38], [39], [40], [41]]. Patients with a low VAT could undergo extra high intensity interval training while awaiting the surgery, thus improving aerobic fitness as was shown by our group [38]. In addition, determination of aerobic fitness could help in the decision making while selecting the appropriate postoperative care, for example, the admission to the intensive care unit. Because cardiopulmonary fitness is by far the most modifiable risk factor, all patients scheduled for major surgeries at our Hepato-Pancreato-Biliary (HPB) surgery department are screened and assessed for low aerobic fitness and are offered a personalized exercise program to improve fitness when needed.

To our knowledge, this is the first case series reporting pancreatic liposarcoma. Our case series represents the third and the fourth cases of coexisting well differentiated liposarcoma and dedifferentiated liposarcoma and the fifth case of pancreatic pleomorphic liposarcoma.

## 6 Conclusion

In this case series, we added three new cases of pancreatic liposarcoma to twenty earlier reported cases in the literature, to increase our understanding of this type of tumor. Surgical resection remains the curative treatment of choice. Pancreatoduodenectomy is considered safe, and it should only be performed when in line with patient physical fitness and preferences. Personalized exercise program to improve fitness is recommended in patients with low aerobic fitness. Retroperitoneal liposarcomas should only be treated in a specialised centres due to their low incidence.

## Author contribution

Study design: ZMA, MWN, JMK

Pathological and imaging data: ZMA, RJH, AC

Writing the original draft: ZMA

Writing and reviewing the manuscript: RJH, AC, BLL, MWN, JMK

## Consent

Written informed consent was obtained from the patient for publication and any accompanying images. A copy of the written consent is available for review by the Editor-in-Chief of this journal on request.

## Ethical approval

This study falls within the scope of the non–WMO system. The Dutch Medical Research with Human Subjects Law is not applicable to this study. All other necessary steps have already been completed. There was no need for approval from the Medical Ethical Evaluation Committee (METc) at our hospital.

## Guarantor

Z.M. Alhulaili

J.M. Klaase

## Funding

This research has not received any funding to be conducted.

## Declaration of competing interest

None.

## Data Availability

Additional data can be requested from the corresponding author.
